# Supplementary material for: Complexome profiling of the Chlamydomonas psb28 mutant reveals TEF5 as an early PSII assembly factor
Source: Plant Cell. 2025 Mar 17;37(6):koaf055. doi: 10.1093/plcell/koaf055 (PMC12164590; doi:10.1093/plcell/koaf055)
Supplement: koaf055_Supplementary_Data [file koaf055_supplementary_data.zip › psb28_interactive.zip!/plots/intensity/qualityPlots/peptides/DVR1_Cre01.g042800.t1.2.html]

### Cre01.g042800.t1.2

Trivial name: DVR1  
SeqCoverage [median]: 46.70  
Mass: 44.62 kDa  
  
 

| Sequence | MissedCleavage | Mass [Da] | wtIntensity | muIntensity | Score |
| --- | --- | --- | --- | --- | --- |
| AVKEGLQGQELGDQAVFGQQ | 1 | 2101.04 | 2641.51 | 14920.12 | 101.49 |
| DSWLIDYTATK | 0 | 1311.63 | 3466.08 | 0.00 | 94.69 |
| DTLEDFFSR | 0 | 1128.51 | 368.50 | 0.00 | 49.42 |
| EDIVKEFPGAEVR | 1 | 1487.76 | 1531.26 | 8013.04 | 77.78 |
| EFPGAEVR | 0 | 903.45 | 0.00 | 2119.56 | 67.98 |
| EGLQGQELGDQAVFGQQ | 0 | 1802.84 | 11893.72 | 36985.24 | 312.31 |
| FGSVLDPASLR | 0 | 1160.62 | 29696.20 | 114554.26 | 202.20 |
| GVYLEDETPGYGK | 0 | 1426.66 | 404.44 | 2388.53 | 123.75 |
| GVYLEDETPGYGKDTLEDFFSR | 1 | 2537.16 | 3119.39 | 0.00 | 66.39 |
| GYNVVAFAR | 0 | 995.52 | 21746.23 | 86627.81 | 157.21 |
| IGKYYATESMLVYDEAR | 1 | 2007.96 | 1109.95 | 3585.40 | 72.82 |
| LFPQLEDSAEFAR | 0 | 1521.75 | 15643.46 | 35662.41 | 155.00 |
| MGREDIVKEFPGAEVR | 2 | 1831.93 | 4571.89 | 17878.18 | 84.14 |
| NSLDVAR | 0 | 773.40 | 20812.46 | 52584.49 | 132.08 |
| SIAGQIDIVK | 0 | 1042.60 | 17887.64 | 69011.23 | 136.99 |
| SIAGQIDIVKK | 1 | 1170.70 | 3838.64 | 23687.70 | 94.77 |
| VLPIGGPSK | 0 | 866.52 | 15197.60 | 42428.28 | 81.19 |
| VLVVGPTGYIGK | 0 | 1201.71 | 34419.08 | 122107.42 | 135.43 |
| YFPVPVALMDGMIGLFDSLAK | 0 | 2283.17 | 786.16 | 0.00 | 106.03 |
| YYATESMLVYDEAR | 0 | 1709.76 | 8747.96 | 43410.53 | 194.17 |
|  |  |  | 197882.2 | 675964.2 |  |
